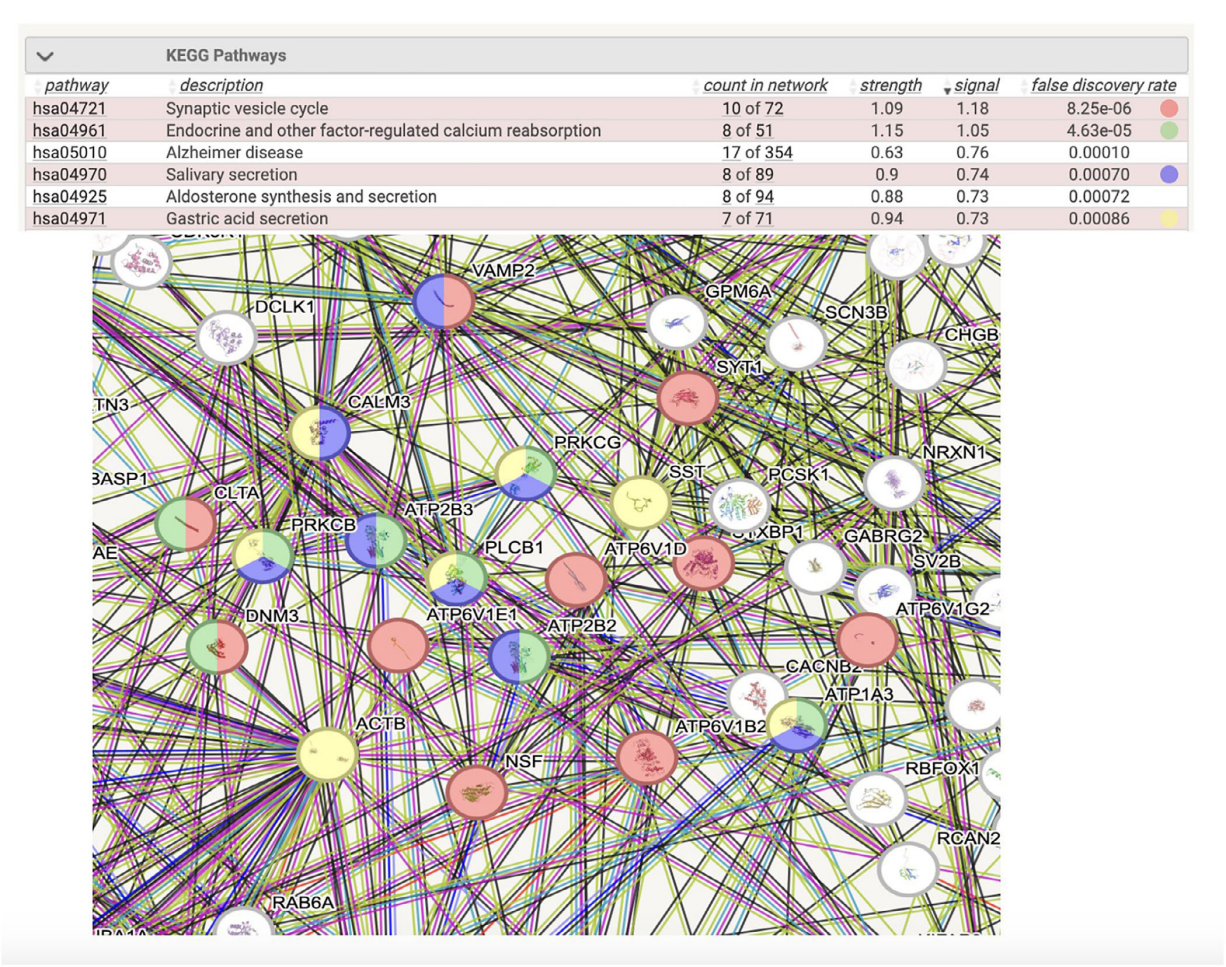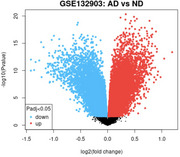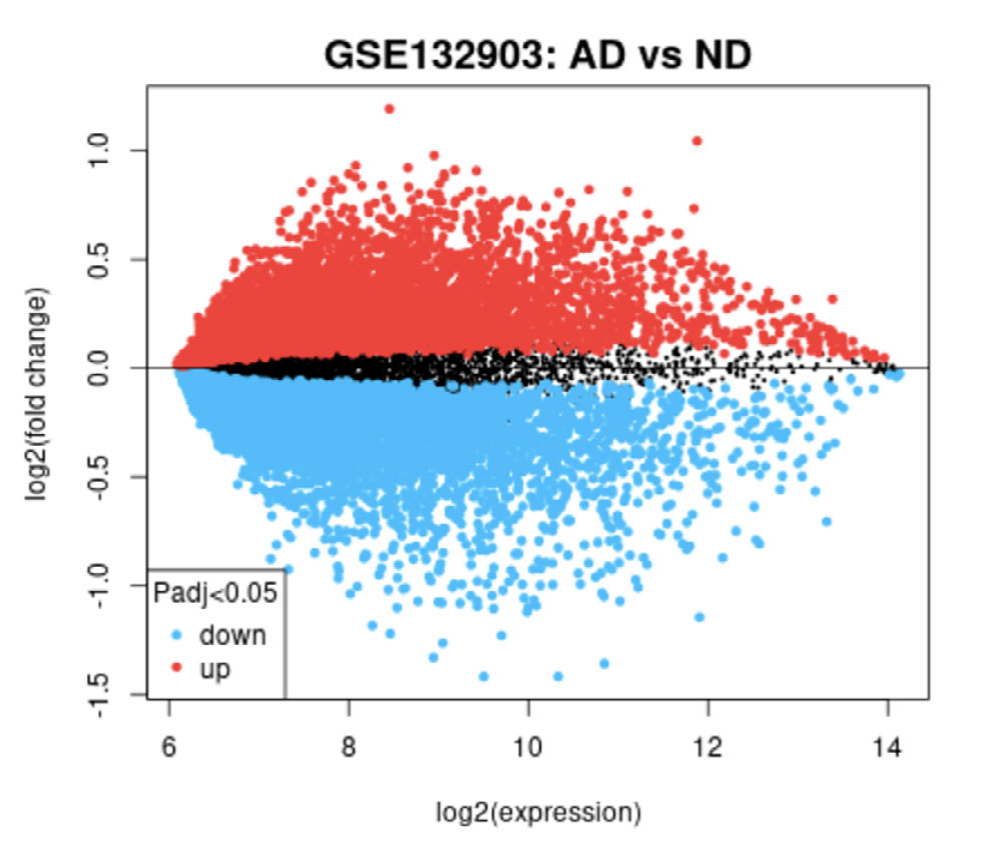# Downregulation of Gastric Acid Secretion Pathways in Alzheimer's Disease: Implications for Autonomic Dysfunction and Gastrointestinal Symptoms

**DOI:** 10.1002/alz70856_101331

**Published:** 2025-12-24

**Authors:** Meher Garg, Inhan Lee

**Affiliations:** ^1^ SIU School of Medicine, Physican pipeline program, Springfield, IL, USA; ^2^ MirCore, Ann Arbor, IL, USA; ^3^ miRcore, Ann Arbor, MI, USA

## Abstract

**Background:**

Alzheimer's Disease (AD) is a neurodegenerative disorder characterized by progressive cognitive decline. While central nervous system pathology is well‐studied, autonomic manifestations, including gastrointestinal (GI) dysfunction, are increasingly recognized. Gastric acid secretion and intestinal motility is often impaired in AD patients. However, the molecular mechanisms driving this dysregulation remain unclear. This study investigates pathways associated with gastric acid secretion in AD, aiming to uncover potential links to autonomic dysfunction and GI symptoms.

**Method:**

Gene Expression Omnibus (GEO) dataset GSE132903, using RNA expression of middle temporal gyrus comprising 97 AD patients and 98 non demented (ND) controls, were analyzed. Differential expression was performed using GEO2R, followed by pathway enrichment analysis through STRING‐DB. Kyoto Encyclopedia of Genes and Genomes (KEGG) pathways related to gastric acid secretion and autonomic regulation were examined, with functional annotations obtained from GeneCards. Statistical analysis and heatmap visualizations were conducted in R/R Studio.

**Results:**

Differential RNA expression analysis revealed significant downregulation of several pathways linked to gastric acid secretion and autonomic regulation in AD group compared to controls. Notably, the Gastric Acid Secretion pathway (hsa04971) had a false discovery rate (FDR) p value of 0.00086, strength of 0.94, and count in network of 7/71. The synaptic vesicle cycling pathway (hsa04961) had a FDR of 8.25e6, strength of 1.09, and count in network of 10/72. The salivary secretion (hsa04925) had a FDR of 0.00070, strength of 0.9, and count in network of 8/89. The endocrine calcium reabsorption pathway had a FDR of 4.63e5, strength of 1.15, and count in network of 8/51. These findings point to a disruption in pathways that may contribute to common GI symptoms such as acid reflux and dyspepsia observed in AD patients.

**Conclusion:**

The downregulation of gastric acid secretion and associated autonomic pathways in Alzheimer's disease (AD) points to a wider autonomic dysfunction within AD. These findings underscore the potential role of impaired gastrointestinal and autonomic pathways in AD. Further research is needed, with a focus on early detection and the implementation of therapeutic strategies to manage GI symptoms.